# Efficacy of Oral Cryotherapy in the Prevention of Oral Mucositis Associated with Cancer Chemotherapy: Systematic Review with Meta-Analysis and Trial Sequential Analysis

**DOI:** 10.3390/curroncol28040250

**Published:** 2021-07-29

**Authors:** Ali Hatem Manfi Al-Rudayni, Divya Gopinath, Mari Kannan Maharajan, Sajesh Kalkandi Veettil, Rohit Kunnath Menon

**Affiliations:** 1 School of Postgraduate Studies, International Medical University, Kuala Lumpur 57000, Malaysia; alihatem.manfi@student.imu.edu.my; 2 Department of Oral Diagnostics & Surgical Sciences, School of Dentistry, International Medical University, Kuala Lumpur 57000, Malaysia; divyagopinath@imu.edu.my; 3 Department of Pharmacy Practice, School of Pharmacy, International Medical University, Kuala Lumpur 57000, Malaysia; marikannan@imu.edu.my; 4 Department of Pharmacotherapy, College of Pharmacy, University of Utah, Salt Lake City, UT 84112, USA; Sajesh.Veettil@pharm.utah.edu; 5 Department of Restorative Dentistry, School of Dentistry, International Medical University, Kuala Lumpur 57000, Malaysia

**Keywords:** oral cryotherapy, oral mucositis, chemotherapy, adverse effects, meta-analysis

## Abstract

Background: This review aimed to evaluate the efficacy of oral cryotherapy in the prevention of chemotherapy-induced oral mucositis using meta-analysis and trial sequential analysis, as well as to assess the quality of the results by the Grading of Recommendations, Assessment, Development and Evaluation (GRADE) approach. Methods: A comprehensive search of three databases including Medline, Embase and Central was performed to identify randomized controlled trials that used oral cryotherapy for the prevention of chemotherapy-induced oral mucositis. The primary outcome was the incidence of oral mucositis for trials employing oral cryotherapy as the intervention for the prevention of oral mucositis. The meta-analysis was performed using the random-effects model and random errors of the meta-analyses were detected by trial sequential analysis. Results: A total of 14 RCTs with 1577 participants were included in the present meta-analysis. Patients treated with oral cryotherapy were associated with a significantly lower risk of developing oral mucositis of any grade (risk ratio (RR), 0.67 (95% CI: 0.56–0.81, *p* < 0.05)). Findings from the subgroup analyses showed that oral cryotherapy significantly reduced the risk of oral mucositis in patients undergoing bone marrow transplantation (RR 0.69, CI: 0.54–0.89, *p* < 0.05) as well as chemotherapy (RR 0.66, CI: 0.58–0.75, *p* < 0.05). Findings from the trial sequential analysis suggested that the evidence on oral cryotherapy as a preventive intervention for oral mucositis in patients with solid malignancies receiving conventional chemotherapy was conclusive. Conclusion: Oral cryotherapy is effective in preventing oral mucositis in patients undergoing chemotherapy for the management of solid malignancies. The use of oral cryotherapy in preventing oral mucositis in bone marrow transplantation settings showed promising efficacy, but the evidence is not conclusive and requires more high-quality randomized controlled trials.

## 1. Introduction

Oral mucositis (OM) is a common complication of cancer treatment, and the incidence may vary according to the antineoplastic treatment modality. OM causes atrophy and destruction of the mucosal lining of the mouth leading to the formation of ulcers. In cancer patients receiving conventional chemotherapy doses, the incidence of OM varies between 20% and 40%, whereas the incidence of OM in patients undergoing bone marrow transplantation (BMT) can be as high as 80% due to the high doses required to achieve myeloablation [1,2]. In subjects undergoing radiotherapy with or without concomitant chemotherapy for head and neck malignancies, the incidence of oral mucositis is higher than 90% [3,4,5]. OM can significantly affect nutritional status, oral hygiene and quality of life [6]. For those patients receiving chemotherapy prior to BMT, OM has been found to be the single most debilitating complication [7]. Moderate to severe OM has been correlated with systemic life-threatening infections, transplant-related sepsis and mortality during periods of extreme immunosuppression [7,8,9]. The severity of oral mucositis in patients with hematologic malignancies receiving BMT was reported to be directly related to an increased duration of requiring total parenteral and narcotic therapy, the incidence of infections, hospitalization time and inpatient charges [7,8]

A wide range of interventions have been tried and tested for the prevention and treatment of OM [1,2,10]. The Mucositis Study Group of the Multinational Association for Supportive Care in Cancer and the International Society of Oral Oncology (MASCC/ISOO) proposed clinical practice guidelines for the prevention and treatment of mucositis [11]. Oral cryotherapy (oral cooling) is popular as a low-cost, simple intervention in preventing the development of OM and is unlikely to cause side effects. The therapy involves placing of any form of ice (chips, cubes or ice-cold water) in the oral cavity before or during chemotherapy administration [12,13]. Inducing local hypothermia in the oral cavity resulting in vasoconstriction of the oral mucosal blood vessels results in a reduction in blood perfusion to the oral mucosal tissues, which in turn would reduce the concentration of the circulating cytotoxic agents in the oral mucosa. Furthermore, OC can also reduce the rate of metabolism in the oral epithelium, which may contribute to reducing the risk of inflammation.

Predictably, the prophylactic mechanism of OC is effective when used with antineoplastic regimens that include agents with short half-life such as 5-FU, methotrexate and HD-melphalan. OC has been shown to be most effective in preventing OM with bolus administration of these agents [14]. During bolus administration, the duration for peak plasma concentration of the cytotoxic agent is short, and thus the duration of OC would be sufficient to limit the reach of the cytotoxic agent to the oral mucosa tissue. The effectiveness of OC in OM may be limited to chemotherapeutic agents, and there is no evidence that OC would be effective in preventing radiotherapy-induced OM.

Several randomized controlled trials and published systematic reviews based on RCTs have suggested that OC is effective in preventing OM in patients undergoing chemotherapy. However, when a meta-analysis includes only a small number of trials, random errors may often lead to a misleading conclusion [15]. This emphasizes the importance of updating the summary of effects of OC on the prevention of OM using recently published trials and considering the risks of random errors. While updating the current evidence, evaluating the efficacy of oral cryotherapy in the prevention of OM is important in supporting its recommendation as a preventive therapy in the management of OM. Evaluating the conclusiveness of the evidence is equally important. Hence, we conducted this study to summarize and synthesize the clinical evidence available from RCTs on the efficacy of OC in the prevention of OM using a meta-analysis coupled with trial sequential analysis (TSA).

## 2. Material and Methods

### 2.1. Study Design

A systematic review and meta-analysis of the efficacy of OC in the prevention of OM in patients undergoing cancer treatment was performed according to the general principles of the Cochrane Handbook for Systematic Reviews of Interventions and was reported according to the Preferred Reporting Items for Systematic Reviews and Meta-Analyses (PRISMA) extension statement [16].

The protocol for the systematic reviews was registered in the international prospective register of systematic reviews PROSPERO ID: CRD42020159547.

### 2.2. Data Resources and Search Strategy

Relevant studies were identified through a systematic search of the Medline, Embase and Cochrane Central Register of Controlled Trials databases from inception until 7 June 2020 by using subject headings and free-text terms. In addition, a search in the published systematic reviews was conducted for additional studies. The search strategy was developed in Medline, and it was applied to other databases. A detailed description of the search strategy is provided in Table S1.

### 2.3. Study Selection

Inclusion criteria.

Studies included in the systematic review were RCTs that met the following inclusion criteria: 

P: patients who were undergoing chemotherapy.

I: oral cryotherapy in any form.

C: control groups received placebo, no treatment, or other active interventions.

O: Primary outcome was the incidence of OM. Secondary outcomes assessed were duration of hospitalization, pain scores and the need for analgesics.

### 2.4. Data Extraction and Quality Assessment

Independent screenings of the titles and abstracts were performed by two authors (AHM and RKM), subsequently followed by full-text readings. Ineligible studies were excluded as per previously specified exclusion criteria (Table S2). Data extraction was performed independently and in duplicate by two reviewers (AHM and RKM) into a data extraction form and was categorized into the following sections: study characteristics, population characteristics, intervention characteristics and outcome definitions and measures. For all outcomes, intention to treat (ITT) analysis was performed [17]. Risk of bias assessment was performed independently by two reviewers (AHM and RKM) using the revised Cochrane risk of bias tool (ROB 2.0) [18].

### 2.5. Data Synthesis

Meta-analysis was performed with the DerSimonian and Laird random-effects model to estimate pooled risk ratios and 95% confidence intervals incorporating heterogeneity within and between studies [18] with Stata version 15.0 (StataCorp, College Station, TX, USA). If a direct comparison was based on two or more studies, heterogeneity between trials was assessed by considering the I^2^ statistics; an I^2^ estimate of ≥50% was interpreted as evidence of substantial levels of heterogeneity [19]. Publication bias and small study effects were assessed using funnel plot asymmetry testing and Egger’s regression test, respectively [20]. A sensitivity analysis was carried out by excluding trials at high risk of bias and subgroup analyses were carried out based on the type of malignancy and cancer treatment protocol.

The risks of random errors were assessed by performing the trial sequential analysis (TSA) using a TSA software package (available at http://www.ctu.dk, accessed on 16 February 2021), which combines information size estimation for meta-analysis (cumulated sample size of included trials) with an adjusted threshold for statistical significance in the cumulative meta-analysis [21]. TSA provides the necessary sample size for the meta-analysis and boundaries that determine whether the evidence in our meta-analyses is reliable and conclusive [15,21]. The quality of evidence was rated using the Grading of Recommendation, Assessment, Development and Evaluation (GRADE) approach [22].

## 3. Results

### 3.1. Study Selection

**Figure 1:**
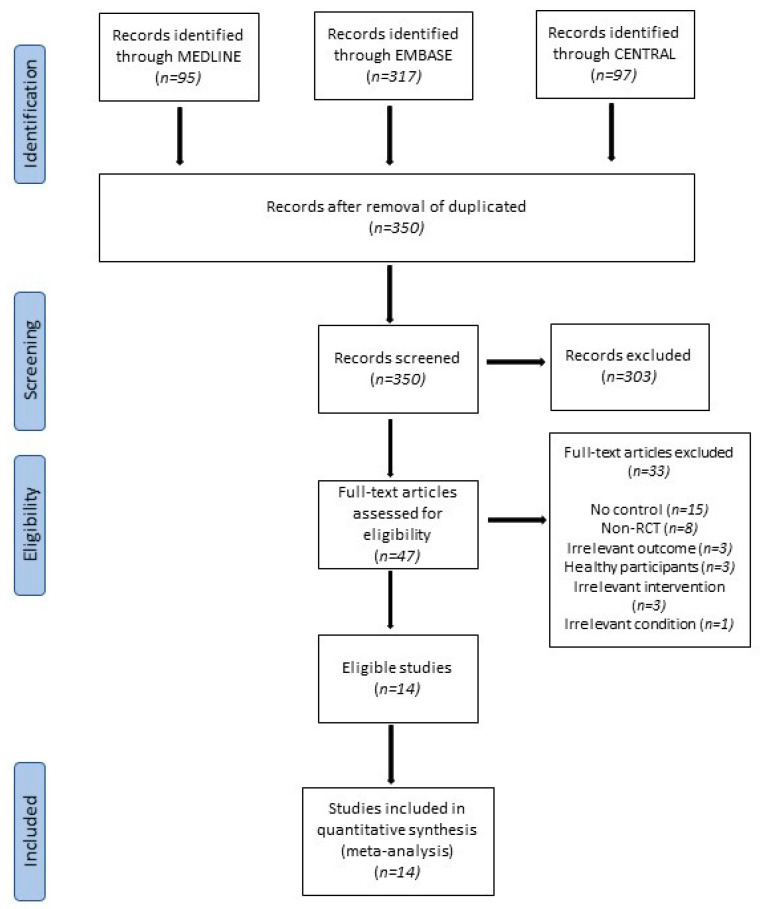
PRISMA flowchart illustrating the study screening and selecting process.

The detailed flow of selection of studies (PRISMA flowchart) is provided in Figure 1. The electronic searches of selected databases retrieved 509 references that were narrowed down to 350 upon removal of the duplicates. After the title and abstract screening, we removed 303 studies. Out of the remaining 47 studies, only 14 studies were selected based on the inclusion criteria (Table 1), and the remaining 33 were excluded because of specific reasons (Table S2).

### 3.2. Characteristics of the Included Studies

The characteristics of the studies are provided in detail in Table 1. A total of 14 studies were included in the final quantitative analysis, which was composed of a total of 1577 participants. Among these, 13 studies consisted of parallel RCTs and 1 was a crossover trial. The RCTs were published in English between 1991 and 2019. Two RCTs were reported from China [23,30], one from Canada [28], three from Italy [24,33,35], one from Sweden [32], one from Turkey [27], one from Denmark [31], two from Iran [25,29] and three from the US [34,36,37]. The number of randomized participants in the included studies ranged from 33 to 220. Four studies compared OC to a normal saline rinse [25,31,34,37], three studies compared OC to oral care [23,27,28] and one study compared OC to leucovorin [30]. Placebo was utilized in six studies [24,29,31,32,35,36]. Among the studies included in our analysis, six studies included participants with mainly solid malignancies with most cancers including colorectal, gastrointestinal and breast cancer. The management of solid malignancies mainly involved fluorouracil (5FU). The rest of the included studies were involved with hematological malignancies. Most of the studies evaluated the incidence of mucositis and its severity grade as the primary outcome. Nine studies used the WHO 0 to 4 scale or an adjusted scale based on this scale. The National Cancer Institute common toxicity criteria (NCI-CTC) 0 to 4 scale was utilized by five studies. Both scales are similar, reliable and include both subjective and objective parameters. One study used the Oral Mucositis Assessment Scale (OMAS) [32].

### 3.3. Risk of Bias Analysis

All the studies were observed to carry out proper sequence generation; thus, the risk of bias that might arise from this domain was assessed as low. Half of the studies did not report the methods utilized to conceal the allocation process; therefore, the risk of bias was assessed as unclear for this domain. Blinding of participants and personnel did not take place in any of the included studies as it is not feasible to perform blinding for OC. Therefore, all the 14 studies were assessed as high risk of bias for this domain. Blinding of the outcome assessment was also not feasible due to the subjective elements involved in the OM assessment scales. Only two studies [30,32] possessed high risk of selective reporting. The risk of attrition bias was low in the majority of the included studies as only two studies possessed a high risk of attrition bias [25,31]. The overall risk of bias assessment was high across all the 14 included studies (Figure S1).

### 3.4. OC Efficacy in Preventing OM (Any Grade)

**Figure 2:**
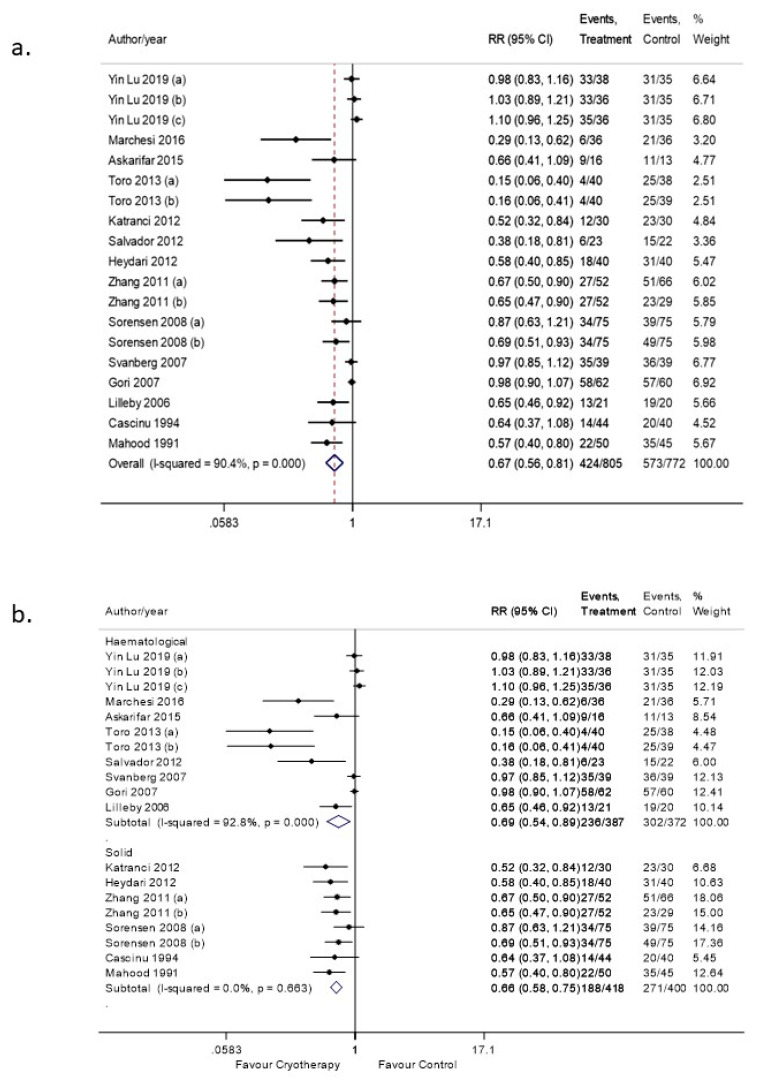
(**a**) Forest plot illustrating pooled data on the effects of OC on the incidence of OM (any grade). (**b**) Forest plot illustrating subgroup analysis according to type of malignancy.

Meta-analysis was performed by compiling all grades of OM as well as subgrouping the studies according to the grade of OM (any grade, moderate-severe and severe). The risk of OM of any grade in the OC group was significantly lower than that in the control group, with a risk ratio (RR) of 0.67 (95% CI: 0.56–0.81, *p* < 0.05). High heterogeneity was identified between the studies, with an I^2^ of 90% indicating a wide variation among the studies (Figure 2a). These variations include the age differences between studies, type of malignancy, type of cancer treatment regimens, control characteristics, duration and timing of OC. Additionally, the Funnel plot asymmetry test illustrated publication bias (Figure S2), which was verified by Egger’s test as well (Table S3).

#### 3.4.1. Sensitivity Analysis

Since the evidence of small study effects was observed for the primary outcome, we performed a sensitivity analysis by excluding small size studies (<25th percentile) to determine the robustness of the findings. A RR of 0.79 (95% CI: 0.68–0.92, *p* < 0.05) suggesting the superiority of OC to prevent OM when compared to the control group was observed. However, high heterogeneity (I^2^ = 84.6%) was detected (Figure S3).

#### 3.4.2. Subgroup Analyses

To explore the heterogeneity, we performed subgroup analyses based on the type of malignancy. Patients in the hematological malignancies subgroup underwent Bone marrow transplantation (BMT) with a mainly high dose melphalan conditioning regimen and patients in the solid malignancies subgroup received conventional chemotherapy with mainly 5-FU based regimens (Figure 2b). For the hematological malignancies subgroup, the level of heterogeneity remained high (I^2^ = 92.8%). However, the I^2^ value for solid malignancies subgroup dropped to 0%.

### 3.5. OC Efficacy in Preventing OM (Moderate-Severe Grade)

**Figure 3:**
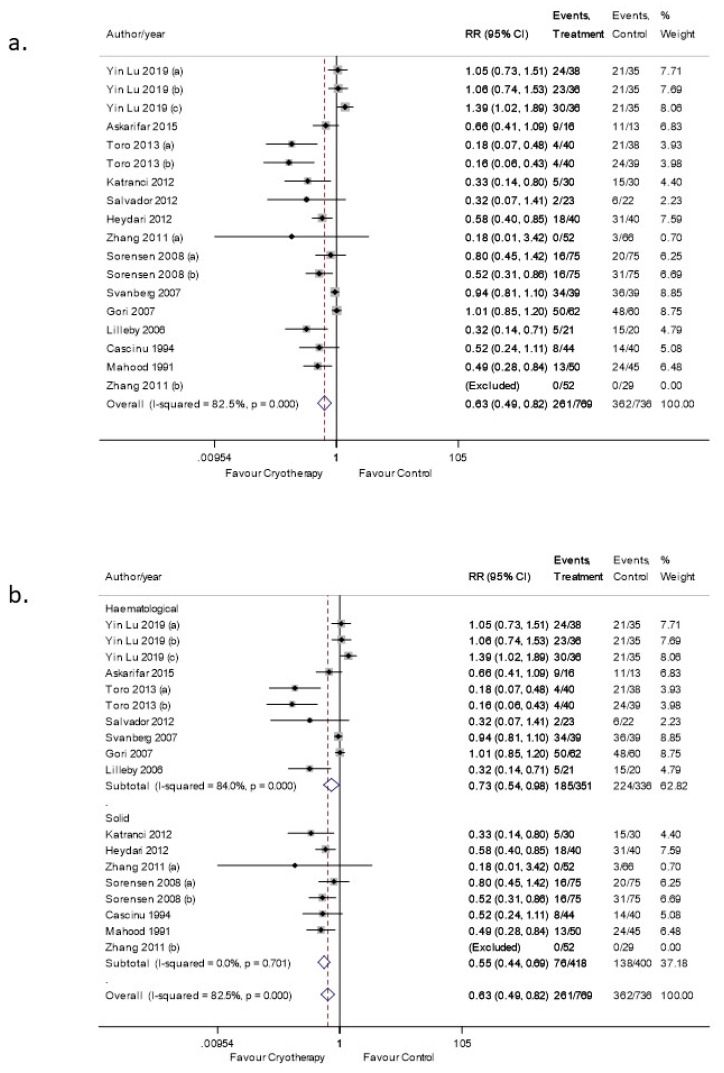
(**a**) Forest plot illustrating the pooled data on the effects of OC on the incidence of OM (moderate-severe). (**b**) Forest plot illustrating subgroup analysis of the effects of OC on the incidence of moderate-severe OM based on the underlying malignancy.

OC significantly reduced the risk of moderate-severe OM with an RR 0.63 (95% CI: 0.49–0.82. *p* < 0.05) (Figure 3a). Nevertheless, an I^2^ value of 82.5% indicated high heterogeneity. The funnel plot asymmetry test confirmed the presence of publication bias (Figure S4), which was verified by Egger’s test as well (Table S4).

#### 3.5.1. Sensitivity Analysis

Sensitivity analysis did not affect the level of heterogeneity and I^2^ value remained high with 76% (Figure S5).

#### 3.5.2. Subgroup Analysis

Subgroup analyses were also performed based on the type of malignancy. For the hematological malignancies subgroup, the level of heterogeneity remained high (I^2^ = 84.0%). However, the I^2^ value for solid malignancies sub-type analysis dropped to 0% with an RR 0.55 (95% CI: 0.44–0.69. *p* < 0.05) (Figure 3b).

### 3.6. OC Efficacy in Preventing OM (Severe Grade)

**Figure 4:**
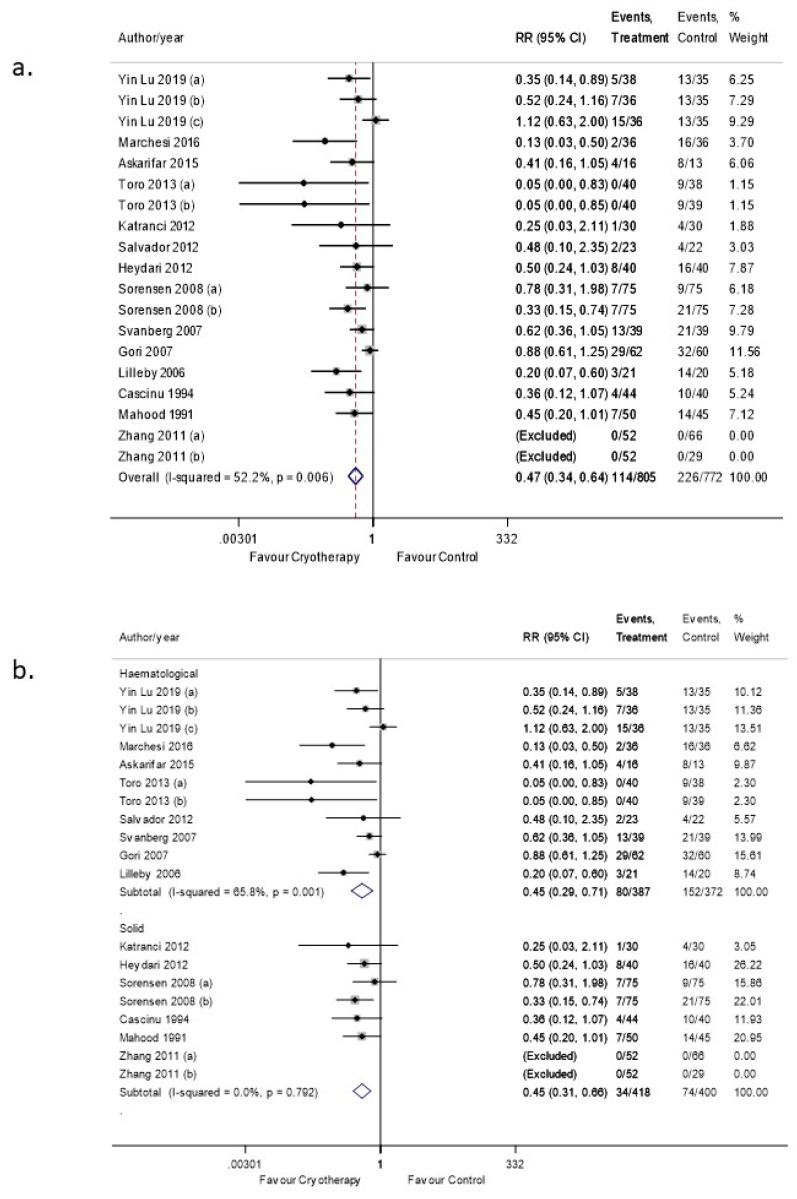
(**a**) Forest plot illustrating the pooled data on the effects of OC on the incidence of OM (severe). (**b**) Forest plot illustrating subgroup analysis of the effects of OC on the incidence of severe OM based on the underlying malignancy.

OC significantly reduced the risk of severe OM with an RR 0.47 (95% CI: 0.34–0.94. *p* < 0.05) (Figure 4a). The level of heterogeneity was relatively lower with an I^2^ of 52.2% indicating moderate heterogeneity. However, heterogeneity of 52.2% may be considered high. The funnel plot detected publication bias (Figure S6), which was verified by Egger’s test as well (Table S5).

#### 3.6.1. Sensitivity Analysis

After excluding trials with a high risk of small study effects, heterogeneity did not decrease as I^2^ remained at 52% (Figure S7)

#### 3.6.2. Subgroup Analyses

Subgroup analyses were performed based on the type of malignancy. For hematological malignancies, the level of heterogeneity remained high (I^2^ = 65.8%). However, the I^2^ value for solid malignancies sub-type analysis dropped to 0% with an RR 0.45 (95% CI: 0.31–0.66. *p* < 0.05) (Figure 4b).

### 3.7. Trial Sequential Analysis for the Incidence of OM (Any Grade) for Those Trials Conducted on the Subgroup of Patients with Solid Malignancies Receiving Conventional Chemotherapy

**Figure 5:**
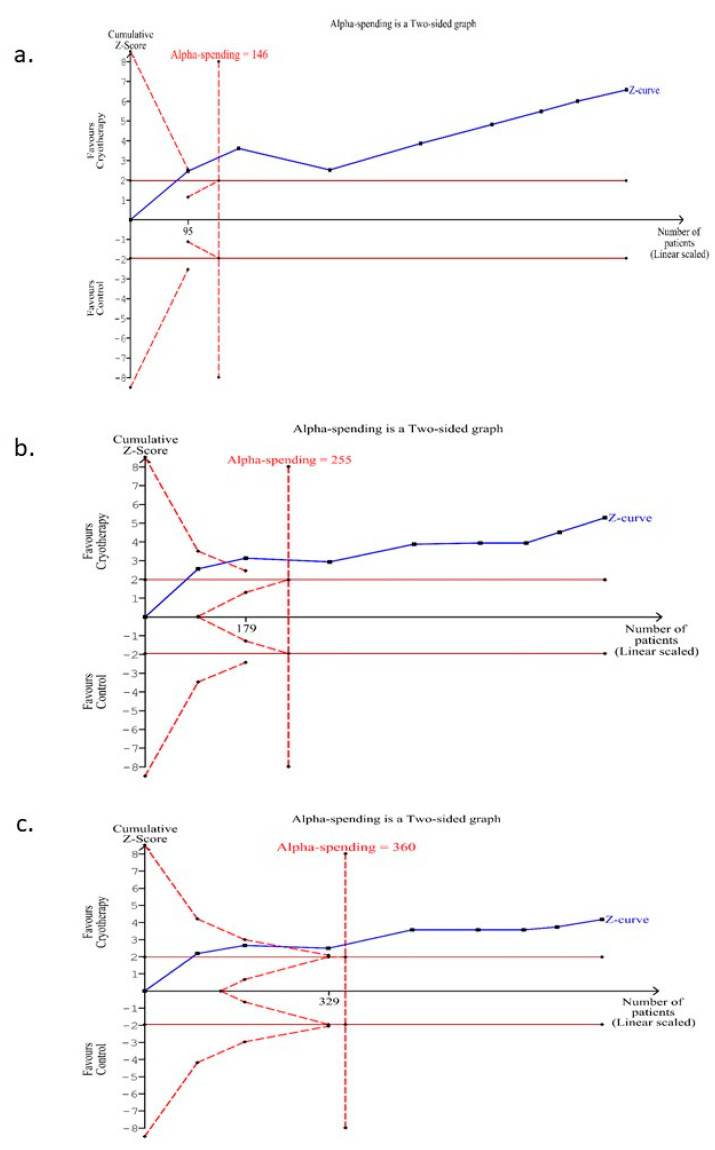
Trial sequential analysis for the OC for solid malignancies subgroup for (**a**) any grade, (**b**) moderate-severe grade and (**c**) severe grade.

Trial sequential analysis for OC compared to control for the incidence of OM (any grade) was undertaken with a type 1 error of 5% and type II error of 20% by using a random-effects model for those trials conducted on the subgroup of patients with solid malignancies receiving conventional chemotherapy. The information size (n = 146) was calculated using an anticipated intervention effect of RR = 0.66 (the intervention effect obtained from the meta-analysis), as shown in Figure 2a, and the control event proportion of 67.75%. The cumulative Z-curve (blue) crossed the conventional (cumulative Z-score between −2 to +2) boundary indicating a significant benefit of the intervention as demonstrated in the meta-analysis (Figure 5a). The number of patients included in the meta-analysis (n = 818) exceeded the required information size. Moreover, the cumulative Z-curve also crossed the alpha-spending boundary (red-dotted line). Hence, the meta-analysis results can be considered conclusive.

### 3.8. Trial Sequential Analysis for the Incidence of OM (Moderate-Severe Grade) for Those Trials Conducted on the Subgroup of Patients with Solid Malignancies Receiving Conventional Chemotherapy

Trial sequential analysis for the OC compared to control for the incidence of OM (moderate-severe grade) was undertaken with a type 1 error of 5% and type II error of 20% by using a random-effects model for those trials conducted on subgroup of patients with solid malignancies receiving conventional chemotherapy. The information size (n = 255) was calculated using an anticipated intervention effect of RR = 0.55 (the intervention effect obtained from the meta-analysis), as shown in Figure 3a. and the control event proportion of 34.5%. The cumulative Z-curve (blue) crossed the conventional (cumulative Z-score between −2 to +2) boundary indicating a significant benefit of the intervention as demonstrated in the meta-analysis (Figure 5b). The number of patients included in the meta-analysis (n = 818) exceeded the required information size. Moreover, the cumulative Z-curve also crossed the alpha-spending boundary (red-dotted line). Hence, the meta-analysis results can be considered conclusive.

### 3.9. Trial Sequential Analysis for the Incidence of OM (Severe Grade) for Those Trials Conducted on the Subgroup of Patients with Solid Malignancies Receiving Conventional Chemotherapy

Trial sequential analysis for the OC compared to control for the incidence of OM (severe grade) was undertaken with type 1 error of 5% and type II error of 20% by using a random-effects model for those trials conducted on chemotherapy-induced OM. The information size (n = 360) was calculated using an anticipated intervention effect of RR = 0.45 (the intervention effect obtained from the meta-analysis), as shown in Figure 4a., and the control event proportion of 18.5%. The cumulative Z-curve (blue) crossed the conventional (cumulative Z-score between −2 to +2) boundary indicating a significant benefit of the intervention as demonstrated in the meta-analysis (Figure 5c). The number of patients included in the meta-analysis (n = 818) exceeded the required information size. Moreover, the cumulative Z-curve also crossed the alpha-spending boundary (red-dotted line). Hence, the evidence obtained from the meta-analysis can be considered conclusive.

GRADE: Summary of evidence.

The GRADE evidence and summary of the findings of our primary outcome is provided in Table S6. Overall, the quality of evidence was graded as moderate.

## 4. Discussion

OM is a prominent adverse event induced by most cancer treatment regimens including chemotherapy, radiotherapy and some targeted therapy agents. OM can complicate and worsen the prognosis of cancer care [38,39]. Several agents have been previously employed for the prevention of OM and cooling of the oral mucosa by cryotherapy is one of the interventions that has shown to be effective in preventing OM induced by chemotherapy. OC was first recommended by MASCC/ISOO in 2004 for the prevention of OM. In the latest MASCC/ISOO guidelines, OC was recommended as a preventive technique for OM induced by 5-FU and melphalan. The recommendation was made with a moderate level of evidence due to the high risk of bias incorporated in the included RCTs [40].

This systematic review and meta-analysis investigated the efficacy of OC in the prevention of OM in cancer patients undergoing either conventional chemotherapy or for BMT conditioning. The results of the meta-analysis show that OC significantly reduced the risk of occurrence of any grade, moderate-severe grade, and severe grade OM in cancer patients undergoing chemotherapy. However, a high level of heterogeneity was detected, which could be due to variation in participant-related factors such as age, type of malignancy type and other parameters. We conducted sensitivity analyses for all the grades we studied, because of the severe heterogeneity observed in our meta-analysis. Notably, small study effects were present in our metanalysis because the number of participants was quite low in some RCTs. Although the sensitivity analysis demonstrated the robustness of our results, the heterogeneity for the pooled effect estimates of the included interventions remained significantly high even after removing the trials showing small study effects. Subgroup analysis illustrated analogous results when we stratified the studies based on OM severity, the cancer types, therapeutic management, individual drugs and relative to the controls employed. However, the heterogeneity significantly diminished to 0 for the pooled effect estimates on trials on chemotherapy-induced mucositis. Hence, we proceeded with trial sequential analysis of this evidence to evaluate the conclusiveness of the evidence on the impact of cryotherapy on the estimates on trials on chemotherapy-induced mucositis regardless of the grade.

TSA illustrated that OC is effective in preventing OM in cancer patients with solid malignancies undergoing chemotherapy (mainly 5-FU-based regimens); the evidence provided from the analysis of solid tumors subgroup is considered conclusive with no need for further research. TSA can provide a conclusive result by maximizing the utilization of available data in the literature. However, TSA requires the studies to have a low risk of bias and a low level of heterogeneity between the studies. Nevertheless, we conducted TSA for the solid malignancies subgroup despite the high risk of bias of the trials included in the analysis after considering the impossibility of blinding in cryotherapy trials, as it involves intervention with unconcealable properties like ice. Thus, the results of this meta-analysis consolidate the recommendation of MASCC/ISOO for using OC for the prevention of OM induced by 5-FU [11].

Several studies have investigated additional outcomes other than effectiveness in the prevention of OM. Since additional outcome measures were inconsistent across studies, only few studies provided consistent data eligible for synthesis. These outcomes analyzed included overall pain score, duration of hospitalization in OM patients and the number of patients requiring analgesics, which were also found to be reduced due to use of cryotherapy. Reporting of additional outcomes can often lead to outcome reporting bias because of selective reporting of results on the basis of statistical significance [41,42]. Therefore, the results of these secondary outcomes should be reviewed with caution, as such practices can lead to exaggerated effect estimates implying amplified effectiveness of an intervention [41,42].

In 2004, the Mucositis Study Group of the Multinational Association of Supportive Care in Cancer and International Society of Oral Oncology (MASCC/ISOO) published recommendations for an evidence-based clinical practice for the prevention and management of OM based on systematical evaluation of the peer-reviewed literature on RCTs. The use of cryotherapy for the prevention of OM in patients on 5-fluorouracil treatment was recommended initially in 2013 [43]. In the latest updates of the MASCC/ ISOO mucositis guidelines, a recommendation in favor of oral cryotherapy was provided for preventing OM in patients receiving HSCT conditioned with chemotherapy [11]. These recommendations were mainly based on systematic reviews of the published studies. Our meta-analysis offers further support to these recommendations as our TSA highlights the conclusiveness of the evidence. 

The result of our meta-analysis supports the findings of previous meta-analyses, which concluded that oral cryotherapy is effective in reducing the incidence and severity of OM [44]. The Cochrane meta-analysis published in 2015 also concluded that oral cryotherapy was effective in preventing OM induced by 5-FU treatment for solid tumors and high-dose melphalan for BMT conditioning [45]. However, there was uncertainty regarding the evidence on the preventive role of OC in BMT patients receiving high-dose melphalan conditioning due to the wide confidence interval. Our meta-analysis incorporates new trials conducted after 2015, and hence presents the most recent and updated pooled effect estimate on the HSCT with a narrow confidence interval, highlighting the efficacy of oral cryotherapy in preventing OM in BMT patients as well. Moreover, TSA emphasizes that the evidence regarding chemotherapy is conclusive. To the best of our knowledge, TSA has never been attempted before to assess the efficacy of oral cryotherapy for OM prevention.

The main inconsistency among the studies that evaluated the prophylactic effect of OC was the duration of cryotherapy treatment. The duration of the OC in the included studies varied from 5 min to the entire session of chemotherapy. A recent RCT, which compared 2 h cryotherapy with 6 h on HSCT patients, reported that 2 h is as efficacious as the 6 h protocol. [46]. Furthermore, Johansson et al. conducted a similar study to compare a longer duration of 6–7 h to 2 h [13] of OC, and they did not report significant differences in OM incidence between the two groups. The impact of time of initiation of OC was investigated by Lu 2019, who reported that there was an increase of OM incidence in the group where OC was started from the midpoint of the chemotherapy session [23]. However, the increase was not significant (*p* > 0.05) [23]. Moreover, additional protocols with cryotherapy beginning 5 min before the procedure as well an entire session of 60 min before were also tried [25].

There was a notable lack of studies on pediatric patients. However, based on the available evidence, the pediatric oncology group (Ontario) recommends the use of oral cryotherapy in children receiving CT or HSCT [47]. Considering the physical nature of this therapy, pediatric patients’ adherence can be improved by using flavored popsicles or frozen smoothies [48]. The pediatric oral mucosa is biologically different from the adults’ oral mucosa. Therefore, additional trials are recommended to explore the feasibility of this preventive option in children. 

All the studies included in the trials possess a high risk of bias, mainly because blinding was not performed in all the included studies. However, this shortcoming is justifiable as it is not possible to blind the participants from the cold sensation of ice. Nonetheless, the overall GRADE concluded that the evidence was of moderate quality. Though effective with chemotherapy-induced OM, the proposed mechanism of action of oral cryotherapy in preventing OM makes it highly unlikely that it would be effective in preventing OM induced by chemotherapy when combined with radiotherapy. The MASCC/ISOO guidelines recommended benzydiamine mouthwash, palifermin and LLLT for the prevention of radiotherapy-induced OM. Additionally, the new under-investigation agent Avasopasem (GC4419) is showing moderate efficacy in chemoradiotherapy-induced OM in phase II trials [49].

## 5. Conclusions

It can be concluded that, OC is effective in preventing OM in patients undergoing chemotherapy regimens for the management of solid malignancies and serves as strong evidence to support MASCC/ISOO guidelines to use OC for the prevention of 5-FU induced OM. The use of OC in preventing OM in BMT settings showed a promising efficacy, but the evidence is not conclusive and requires further research with robust RCTs.

## Figures and Tables

**Table 1:** Characteristics of studies included in meta-analysis.

Author	Country	Design	Age Groups	No.	Cooling Method	Control	Malignancy Type	Antineoplastic Therapy	OC Duration
Lu 2019 [23]	China	Parallel RCT, 4 arms	Adults	160	Ice cubes	CHX	Hematological	BMT, BUCY	Entire session
Marchesi 2017 [24]	Italy	Parallel RCT, 2 arms	Adults	72	Ice chips	PLACEBO	Hematological	BMT, MELPH	15−30 min
Askarifar 2016 [25]	Iran	Parallel RCT, 2 arms	Adults	33	Nylon ice packs	NS	Hematological	BMT, MELPH	15−30 min
Toro 2013 [26]	US	Parallel RCT, 3 arms	Adults	78	Crushed ice	NS	Hematological	BMT, MELPH	105 min
Katrancı 2012 [27]	Turkey	Parallel RCT, 2 arms	N/A	60	Ice chips	BOC	Solid	CT, 5FU	30 min
Salvador 2012 [28]	Canada	Parallel RCT, 2 arms	Adults	46	Ice chips	BOC	Hematological	BMT, HD-MELPH	60 min
Heydari 2012 [29]	Iran	Parallel RCT, 2 arms	Adults	80	Ice chips	BOC	Solid	CT, MAYO, CAF, CMF	Whole session
Zhang 2011 [30]	China	Parallel RCT, 4 arms	Mixed	147	Iced water	PLACEBO	Solid	CT, MTX	N/A
Sorensen 2008 [31]	Denmark	Parallel RCT, 3 arms	Adults	225	Crushed ice	LV	Solid	CT, 5FU	45 min
Svanberg 2007 [32]	Sweden	Parallel RCT, 2 arms	Adults	78	Ice chips	NS/CHX	Hematological	BMT, HD-MELPH	Entire session
Gori 2007 [33]	Italy	Parallel RCT, 2 arms	Adults	130	Ice chips/ Popsicles	BOC	Hematological	BMT, CT/RT	60 min
Lilleby 2006 [34]	US	Parallel RCT, 2 arms	Adults	41	Ice chips	PLACEBO	Hematological	BMT, HD-MELPH	6 h
Cascinu 1994 [35]	Italy	Parallel RCT, 2 arms	Adults	84	Ice chips	PLACEBO	Solid	CT, 5FU	35 min
Mahood 1991 [36]	US	Crossover RCT, 2 arms	Adults	95	Ice chips	PLACEBO	Solid	CT, 5FU	35 min

CHX: chlorohexidine. BMT: bone marrow transplantation. BUCY: Busulfan, Cytarabine, Cyclophosphamide. MELPH: melphalan. NS: normal saline. MTX: methotrexate. 5FU: 5-fluorouracil. MAYO 5-FU bolus+ Leucovorin (low dose). LV: leucovorin. CAF: cyclophosphamide, doxorubicin, 5-FU. CMF: cyclophosphamide, methotrexate, fluorouracil. BOC: basic oral care. CT: chemotherapy. RT: radiotherapy. WHO-OTS: World Health Organization oral toxicity scale. NCI-CTCAE: National Cancer Institute common toxicity criteria adverse events. PROM: patient-reported oral mucositis. QoL: quality of life.

## Data Availability

No new data were created or analyzed in this study. Data sharing is not applicable to this article.

## Supplementary Materials

The following are available online at https://www.mdpi.com/article/10.3390/curroncol28040250/s1, Figure S1: Risk of bias analysis for included studies; Figure S2: Funnel plot asymmetry test for included studies in oral cryotherapy meta-analysis (OM any grade); Figure S3: Forest plot for sensitivity analysis illustrating subgroup analysis of the effects of OC on the incidence of OM (any grade) in patients based on the underlying malignancy; Figure S4: Funnel-plot illustrating the publication bias for oral cryotherapy studies (OM moderate-severe grade); Figure S5: Forest plot for sensitivity analysis illustrating subgroup analysis of the effects of OC on the incidence of OM (moderate-severe grade) in patients based on the underlying malignancy; Figure S6: Funnel-plot illustrating the publication bias for oral cryotherapy studies (OM severe grade); Figure S7: Forest plot for sensitivity analysis illustrating subgroup analysis of the effects of OC on the incidence of OM (severe) in patients based on the underlying malignancy; Table S1: Search terms and results oral cryotherapy; Table S2: Excluded articles for oral cryotherapy with reason; Table S3: Egger’s regression test for OC in preventing OM (any grade); Table S4: Egger’s regression test (OM moderate-severe grade); Table S5: Egger’s regression test (Severe grade); Table S6: summary of findings and level of evidence for OC meta-analysis.
