# Supplementary material for: Efficacy of Oral Cryotherapy in the Prevention of Oral Mucositis Associated with Cancer Chemotherapy: Systematic Review with Meta-Analysis and Trial Sequential Analysis
Source: Curr Oncol. 2021 Jul 29;28(4):2852–67. doi: 10.3390/curroncol28040250 (PMC8395421; doi:10.3390/curroncol28040250)
Supplement: Supplementary file 1 [file curroncol-28-00250-s001.zip › curroncol-1250628-supplementary.pdf]

Table S1: Search terms and results oral cryotherapy

#	**Keyword**	Medline	Embase	CENTRAL
1	exp NEOPLASMS/	3325911	4875367	77595
2	exp LEUKEMIA/	231007	335868	4644
3	exp LYMPHOMA/	170899	324641	3173
4	exp RADIOTHERAPY/	184473	598222	5856
5	exp Antineoplastic agents/	1092432	2344609	54485
6	Bone Marrow Transplantation/	44508	53048	1368
7	neoplasm$.mp.	2805134	874920	79397
8	cancer$.mp.	1804027	3712965	169046
9	(leukaemi$ or leukemi$).mp.	329165	503434	15026
10	(tumour$ or tumor$).mp.	2110490	3381384	78405
11	malignan$.mp.	577527	953635	27680
12	neutropeni$.mp.	44812	130357	14301
13	carcino$.mp.	1032312	1627071	45899
14	adenocarcinoma$.mp.	240394	293463	11190
15	lymphoma$.mp.	245229	343706	12414
16	(radioth$ or radiat$ or irradiat$).mp.	901010	1446902	51221
17	(bone adj marrow adj5 transplant$).mp.	58908	90028	4001
18	chemo$.mp.	763392	1369673	92912
19	or/1-18	5732334	8427225	292944
20	exp STOMATITIS/	16660	53767	1024
21	Candidiasis, Oral/	4676	3890	213
22	stomatitis.mp.	24360	46240	3995
23	mucositis.mp.	10054	18284	3488
24	(oral adj6 mucos$).mp.	23056	34044	3078
25	(mycosis or mycotic).mp.	18418	81913	1489
26	mIAS.ti,ab.	370	544	20
27	or/20-26	78010	188196	9678
28	Cryotherapy/	5074	18623	639
29	cryotherap$.mp.	10298	21896	2150
30	(cold or freez$ or ice).mp.	255226	341679	14941
31	or/28-30	263524	360356	16427
32	random$.ti,ab.	1134276	1553420	956392
33	placebo$.ti,ab.	215062	314382	298344
34	(doubl$ adj blind$).ti,ab.	150707	215598	239421
35	(singl$ adj blind$).ti,ab.	18288	25139	27309
36	allocat$.ti,ab.	119218	154053	75281

37	clinical study/ or clinical trial/ or controlled clinical trial/	549332	1217093	53
38	OR/32-37	1636647	2616621	14941
39	19 and 27 and 31 and 38	95	**317**	97

Table S2 Excluded articles for oral cryotherapy with reason

#	Author/Year	Title	Reason
1	**J Stubner 2019**	Efficacy of higher temperature cryotherapy.	Healthy participants, testing alternative oral cooling technique
2	**J.-E., Johansson 2019**	Cryotherapy as prophylaxis against oral mucositis after high-dose melphalan and autologous stem cell transplantation for myeloma: a randomised, open-label, phase 3, non-inferiority trial	Both groups received oral cryotherapy
3	**J., Walladbegi 2017**	Innovative intraoral cooling device better tolerated and equally effective as ice cooling	Healthy participants, testing alternative oral cooling technique
4	**Y.K., Cho 2017**	Associations of High-Dose Melphalan Pharmacokinetics and Outcomes in the Setting of a Randomized Cryotherapy Trial	Both groups received oral cryotherapy
5	**P.E.D., dos Reis 2016**	Chamomile infusion cryotherapy to prevent oral mucositis induced by chemotherapy: a pilot study	Both groups received oral cryotherapy
6	**L., Leppla 2016**	An oral care self-management support protocol (OrCaSS) to reduce oral mucositis in hospitalized patients with acute myeloid leukemia and allogeneic hematopoietic stem cell transplantation: a randomized controlled pilot study	No cryotherapy
7	**J., Walladbegi 2016**	Comfortable cooling device versus nature	Both groups received oral cryotherapy
8	**A., Svanberg 2015**	Caphosol() mouthwash gives no additional protection against oral mucositis compared to cryotherapy alone in stem cell transplantation. A pilot study	Both groups received oral cryotherapy
9	**D.J., Harris 2008**	Putting evidence into practice: evidence-based interventions for the management of oral mucositis	Review
10	**H.V., Worthington 2007**	Interventions for preventing oral mucositis for patients with cancer receiving treatment	Review
11	**K., Choi 2007**	The effect of oral glutamine on 5-fluorouracil/leucovorin-induced mucositis/stomatitis assessed by intestinal permeability test	Healthy participants, no control

12	**O.K., Arikan 2006**	A prospective randomized controlled trial to determine if cryotherapy can reduce the pain of patients with minor form of recurrent aphthous stomatitis	Non-oncology related stomatitis
13	**S., Nikoletti 2005**	Comparison of plain ice and flavored ice for preventing oral mucositis associated with the use of 5 fluorouracil	Both groups received oral cryotherapy
14	**M., Nottage 2003**	Sucralfate mouthwash for prevention and treatment of 5-fluorouracil-induced mucositis: A randomized, placebo-controlled trial	Both groups received oral cryotherapy
15	**J.E., Clarkson 2003**	Interventions for preventing oral mucositis for patients with cancer receiving treatment	Review
16	**S.H., Okuno 1999**	Alleviation of gastrointestinal mucosal toxicity related to chemotherapy and radiation therapy: The NCCTG experience	Review? No access
17	**P., Plevova 1999**	Prevention and treatment of chemotherapy- and radiotherapy-induced oral mucositis: A review	Review
18	**S.H., Okuno 1999**	Phase III controlled evaluation of glutamine for decreasing stomatitis in patients receiving fluorouracil (5-FU)-based chemotherapy	Both groups received oral cryotherapy
19	**C.L., Loprinzi 1997**	Phase III controlled evaluation of sucralfate to alleviate stomatitis in patients receiving fluorouracil-based chemotherapy	Both groups received oral cryotherapy
20	**P., Fidler 1996**	Prospective evaluation of a chamomile mouthwash for prevention of 5-FU- induced oral mucositis	Both groups received oral cryotherapy
21	**Rocke, L K 1993**	A randomized clinical trial of two different durations of oral cryotherapy for prevention of 5-fluorouracil-related stomatitis.	Both groups received oral cryotherapy
22	**S., Cabrera-Jaime 2018**	Efficacy of Plantago major, chlorhexidine 0.12% and sodium bicarbonate 5% solution in the treatment of oral mucositis in cancer patients with solid tumour: a feasibility randomised triple-blind phase III clinical trial	No cryotherapy
23	**L., Mishra 2017**	Effect of flavored (Honey and tulsi) ice chips on reduction of oral mucositis among children receiving chemo therapy	Both groups received cryotherapy
24	**Fidler P O'Fallon JR 1996**	Prospective evaluation of a chamomile mouthwash for prevention of 5-FU-induced oral mucositis	Both groups received cryotherapy
25	**: Gjurik, Mirjana 2017**	Oral mucositis during melphalan conditioning for autologous transplantation in multiple myeloma: can local cryotherapy help?	NO FULL TEXT ACCESS
26	**Lu 2013**	Oral Cryotherapy For The Prevention Of Mucositis Following Myeloablative Conditioning and Hematopoietic Stem Cell Transplantation	Both groups received cryotherapy
27	**D.W., Sborov 2014**	2-hour cryotherapy effectively reduces severe mucositis associated with high-dose melphalan followed by stem cell rescue: Results from a randomized trial	Both groups received cryotherapy

28	**T., Kamsvag Magnusson 2016**	Oral cryotherapy in children receiving high dose chemotherapy to avoid mucositis - Is it feasible?	Irrleveant outcome, children
29	**E., Papadeas 2007**	Prevention of 5-fluorouracil-related stomatitis by oral cryotherapy: A randomized controlled study	Not RCT
30	**E., Papadeas 2007**	Prevention of 5-fluorouracil-related stomatitis by oral cryotherapy: A randomized controlled study	Not RCT
31	**Shin N 2019**	[The Effects of Oral Cryotherapy on Oral Mucositis, Reactive Oxygen Series, Inflammatory Cytokines, and Oral Comfort in Gynecologic Cancer Patients Undergoing Chemotherapy: A Randomized Controlled Trial]. Shin N, Kang Y. Apr 2019	Primary Outcome: Level of stomatitis measured by Oral assessment guide (OAG)<br><br>Korean no translation
32	**Y., Erden 2017**	Comparison of efficacy of cryotherapy and chlorhexidine to oral nutrition transition time in chemotherapy-induced oral mucositisj	Irrlevant outcome
33	**de Paula Eduardo F 2015**	Efficacy of cryotherapy associated with laser therapy for decreasing severity of melphalan-induced oral mucositis during hematological stem-cell transplantation: a prospective clinical study.	LLLT co-intervention,

Table S3 Egger's regression test for OC in preventing OM (any grade)

Egger's test

Std_Eff	Coef.	Std. Err.	t	P>|t|	[95% Conf. Interval]	
slope	.2147873	.0404778	5.31	0.000	.1293865	.300188
bias	-3.418132	.363481	-9.40	0.000	-4.18501	-2.651254

Table S4 Egger's regression test (OM moderate-severe grade)

Egger's test

Std_Eff	Coef.	Std. Err.	t	P>|t|	[95% Conf. Interval]	
slope	.235639	.104693	2.25	0.040	.0124912	.4587867
bias	-2.504904	.568947	-4.40	0.001	-3.717586	-1.292222

Table S5 Egger's regression test (Severe grade)

Egger's test

Std_Eff	Coef.	Std. Err.	t	P>|t|	[95% Conf.	Interval]
slope	.2803763	.1719694	1.63	0.124	-.0861679	.6469205
bias	-2.310736	.4290062	-5.39	0.000	-3.225141	-1.396331

Table S6: summary of findings and level of evidence for OC meta-analysis

Summary of findings:

Oral Cryotherapy compared to Controls for Prevention of oral mucositis

Patient or population: Prevention of oral mucositis

Setting:

Intervention: Oral Cryotherapy

Comparison: Control

Outcomes	**Anticipated absolute effects*** (95% CI)		Relative effect (95% CI)	№ of participants (studies)	Certainty of the evidence (GRADE)	Comments
	**Risk with Control**	**Risk with Oral Cryotherapy**				
Incidence of oral mucositis (Any grade)	742 per 1,000	**497 per 1,000** (416 to 601)	**RR 0.67** (0.56 to 0.81)	1577 (14 RCTs)	⊕⊕⊕◯ MODERATE	
Incidence of oral mucositis (Moderate-Severe)	492 per 1,000	**310 per 1,000** (241 to 403)	**RR 0.63** (0.49 to 0.82)	1505 (14 RCTs)	⊕⊕⊕◯ MODERATE	
Incidence of oral mucositis (Severe)	293 per 1,000	**138 per 1,000** (100 to 187)	**RR 0.47** (0.34 to 0.64)	1577 (14 RCTs)	⊕⊕⊕◯ MODERATE	

***The risk in the intervention group** (and its 95% confidence interval) is based on the assumed risk in the comparison group and the **relative effect** of the intervention (and its 95% CI).

CI: Confidence interval; **RR:** Risk ratio

GRADE Working Group grades of evidence
High certainty: We are very confident that the true effect lies close to that of the estimate of the effect
Moderate certainty: We are moderately confident in the effect estimate: The true effect is likely to be close to the estimate of the effect, but there is a possibility that it is substantially different
Low certainty: Our confidence in the effect estimate is limited: The true effect may be substantially different from the estimate of the effect
Very low certainty: We have very little confidence in the effect estimate: The true effect is likely to be substantially different from the estimate of effect

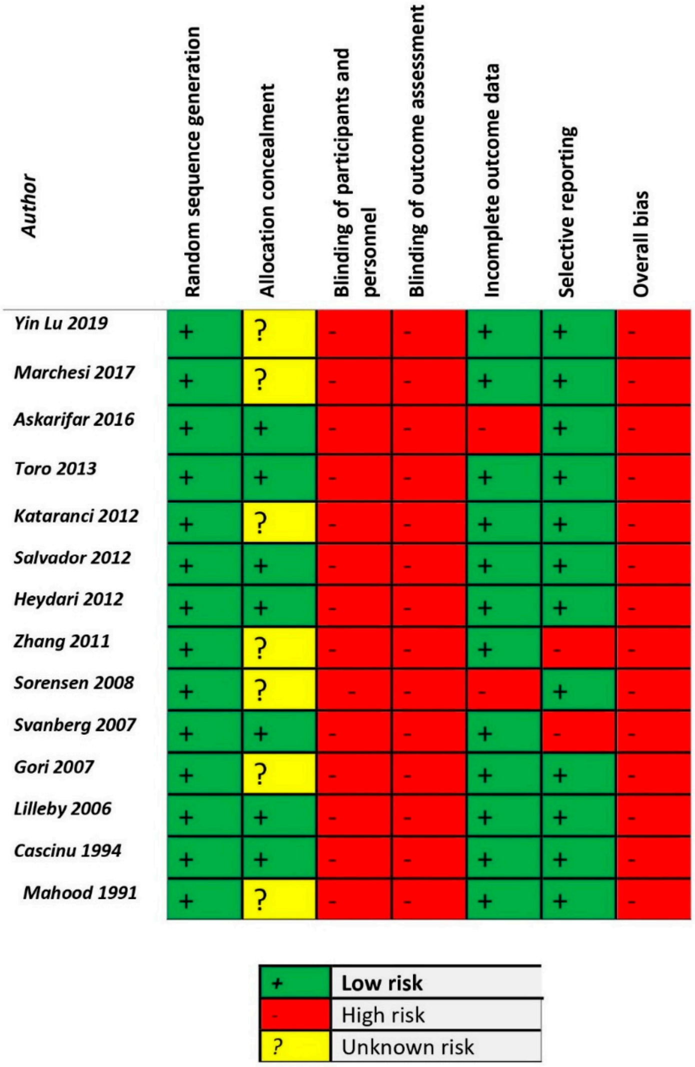

Author	Random sequence generation	Allocation concealment	Blinding of participants and personnel	Blinding of outcome assessment	Incomplete outcome data	Selective reporting	Overall bias
Yin Lu 2019	+	?	-	-	+	+	-
Marchesi 2017	+	?	-	-	+	+	-
Askarifar 2016	+	+	-	-	-	+	-
Toro 2013	+	+	-	-	+	+	-
Kataranci 2012	+	?	-	-	+	+	-
Salvador 2012	+	+	-	-	+	+	-
Heydari 2012	+	+	-	-	+	+	-
Zhang 2011	+	?	-	-	+	-	-
Sorensen 2008	+	?	-	-	-	+	-
Svanberg 2007	+	+	-	-	+	-	-
Gori 2007	+	?	-	-	+	+	-
Lilleby 2006	+	+	-	-	+	+	-
Cascinu 1994	+	+	-	-	+	+	-
Mahood 1991	+	?	-	-	+	+	-

+	**Low risk**
-	High risk
?	Unknown risk

Figure S1. Risk of bias analysis for included studies.

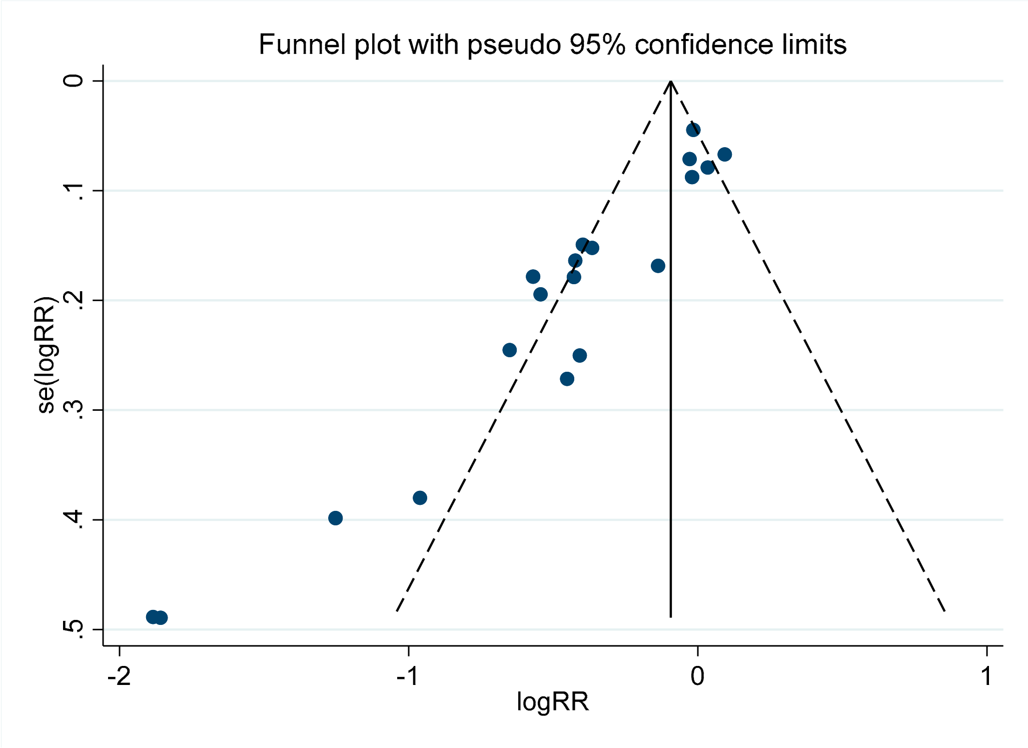

Figure S2 Funnel plot asymmetry test for included studies in oral cryotherapy meta-analysis (OM any grade)

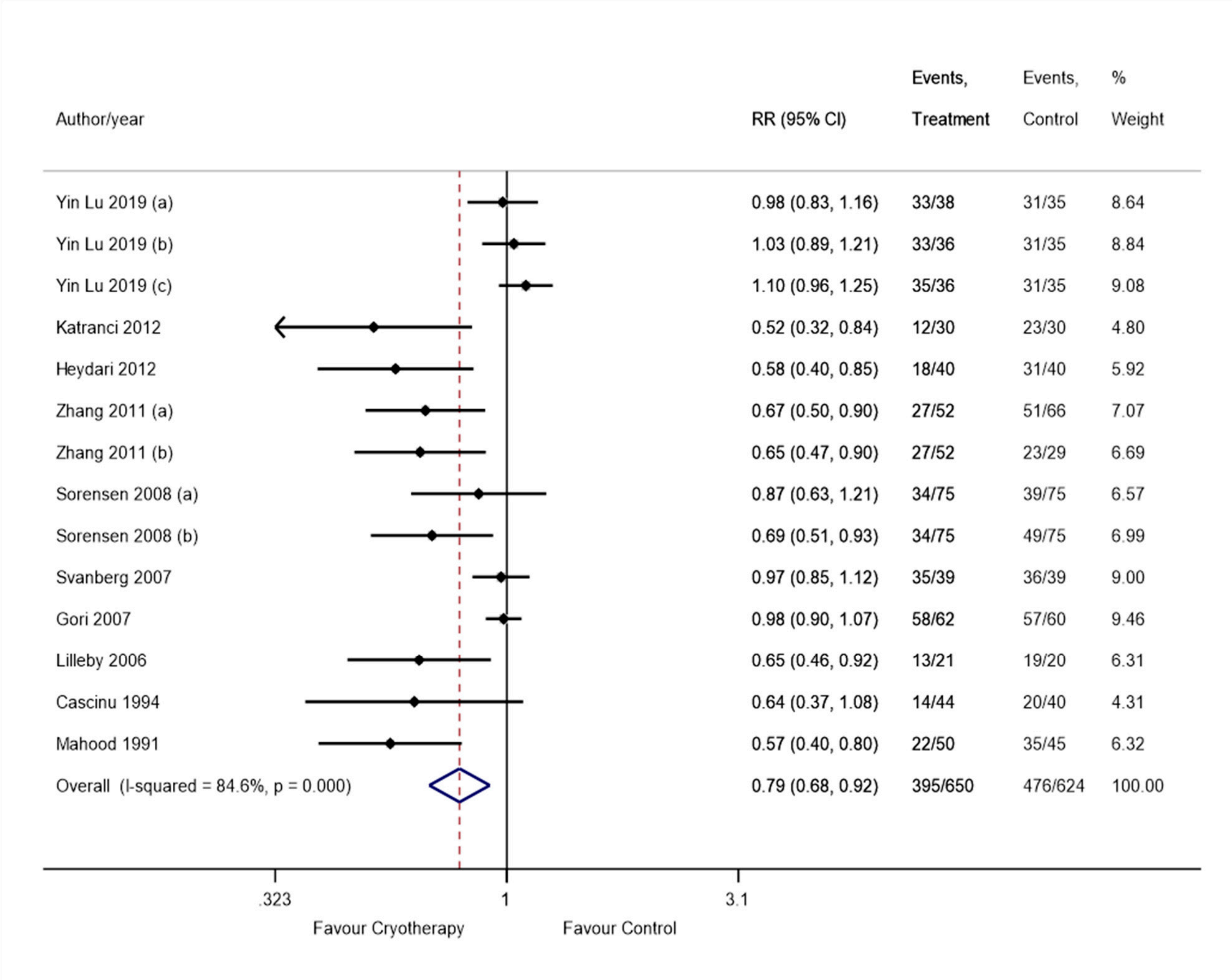
Author/year
RR (95% CI)
Events, Treatment
Events, Control
% Weight
Yin Lu 2019 (a) 0.98 (0.83, 1.16) 33/38 31/35 8.64
Yin Lu 2019 (b) 1.03 (0.89, 1.21) 33/36 31/35 8.84
Yin Lu 2019 (c) 1.10 (0.96, 1.25) 35/36 31/35 9.08
Katranci 2012 0.52 (0.32, 0.84) 12/30 23/30 4.80
Heydari 2012 0.58 (0.40, 0.85) 18/40 31/40 5.92
Zhang 2011 (a) 0.67 (0.50, 0.90) 27/52 51/66 7.07
Zhang 2011 (b) 0.65 (0.47, 0.90) 27/52 23/29 6.69
Sorensen 2008 (a) 0.87 (0.63, 1.21) 34/75 39/75 6.57
Sorensen 2008 (b) 0.69 (0.51, 0.93) 34/75 49/75 6.99
Svanberg 2007 0.97 (0.85, 1.12) 35/39 36/39 9.00
Gori 2007 0.98 (0.90, 1.07) 58/62 57/60 9.46
Lilleby 2006 0.65 (0.46, 0.92) 13/21 19/20 6.31
Cascinu 1994 0.64 (0.37, 1.08) 14/44 20/40 4.31
Mahood 1991 0.57 (0.40, 0.80) 22/50 35/45 6.32
Overall (I-squared = 84.6%, p = 0.000) 0.79 (0.68, 0.92) 395/650 476/624 100.00
.323
1
3.1
Favour Cryotherapy
Favour Control

Figure S3. Forest plot for sensitivity analysis illustrating subgroup analysis of the effects of OC on the incidence of OM (any grade) in patients based on the underlying malignancy.

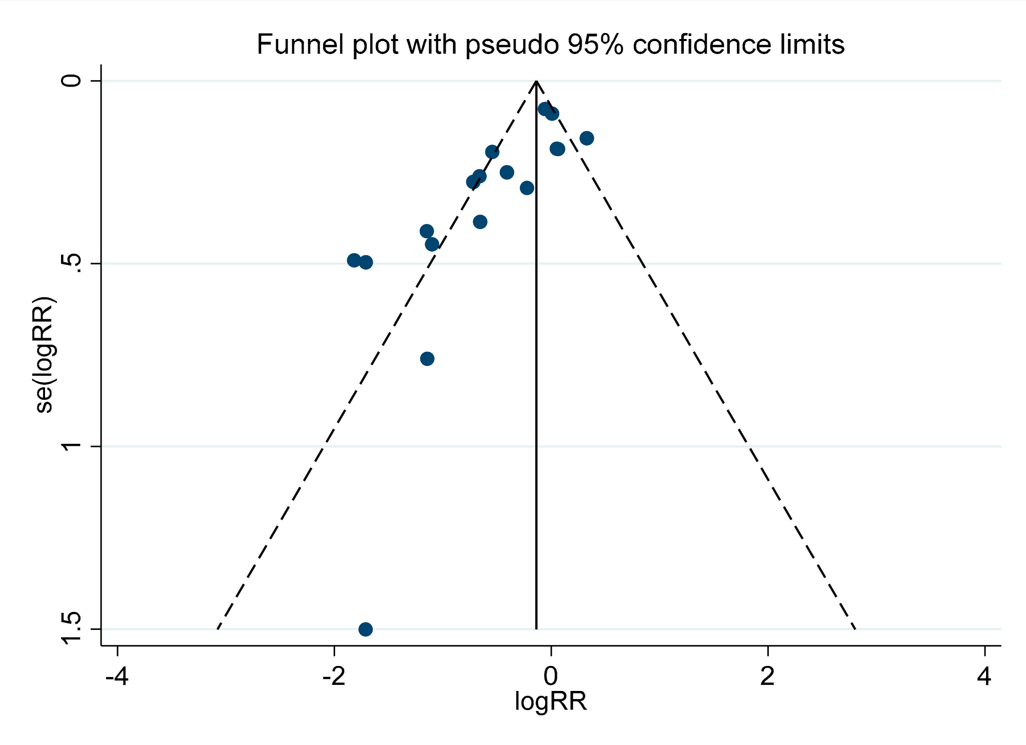

Figure S4: Funnel-plot illustrating the publication bias for oral cryotherapy studies (OM moderate-severe grade)

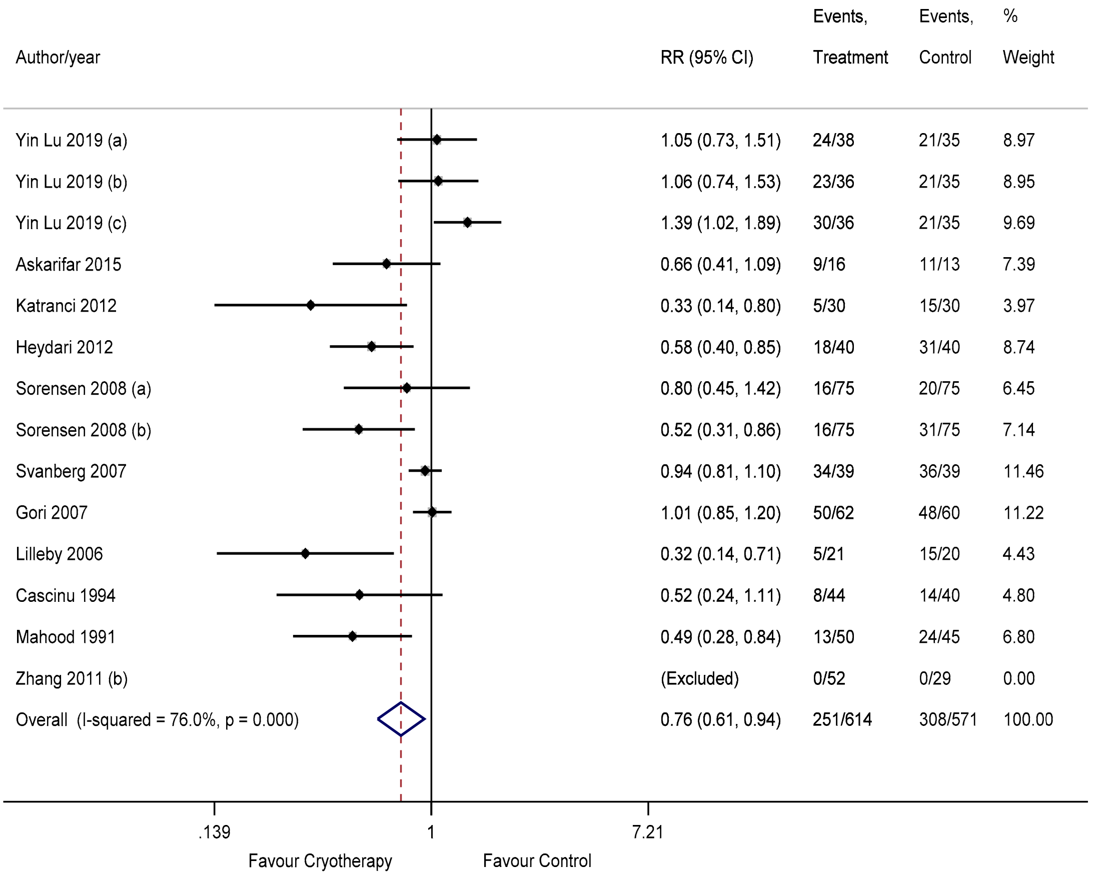

Figure S5: Forest plot for sensitivity analysis illustrating subgroup analysis of the effects of OC on the incidence of OM (moderate-severe grade) in patients based on the underlying malignancy.

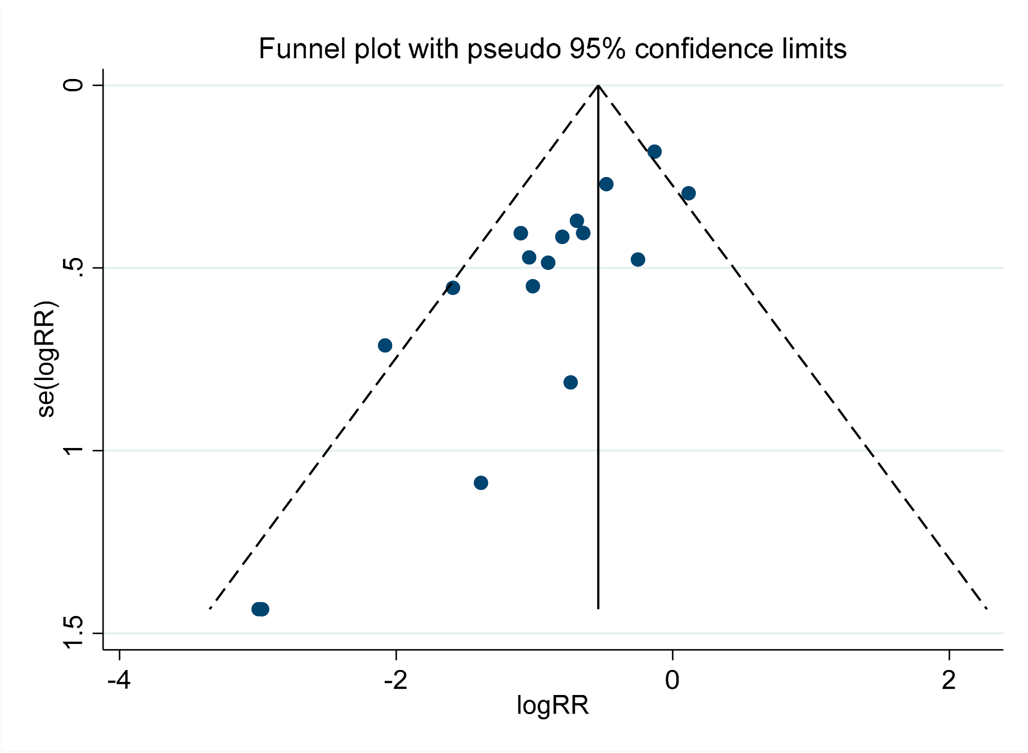

Figure S6: Funnel-plot illustrating the publication bias for oral cryotherapy studies (OM severe grade)

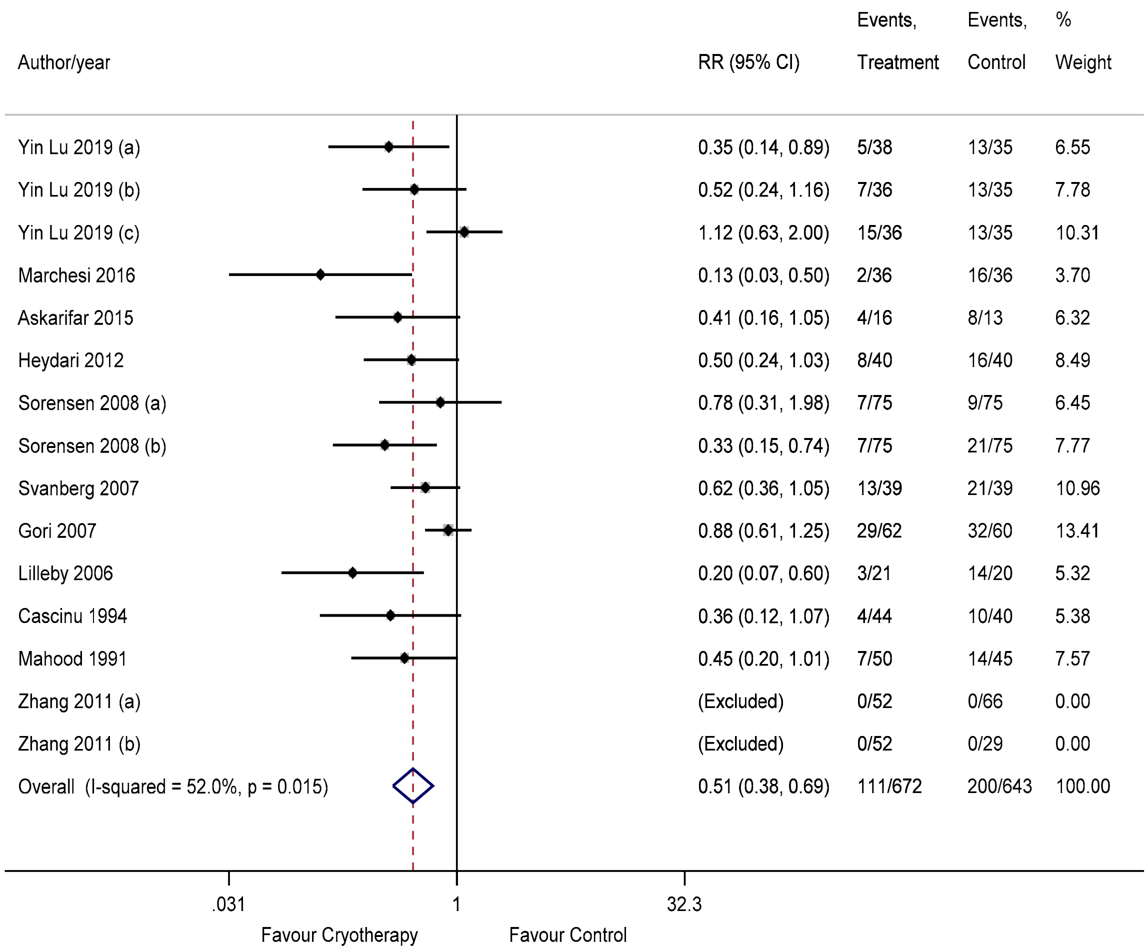

Figure S7: Forest plot for sensitivity analysis illustrating subgroup analysis of the effects of OC on the incidence of OM (severe) in patients based on the underlying malignancy.
